# Unusual Liver Mass in an Immunocompetent Adult: A Case Report and Literature Review

**DOI:** 10.7759/cureus.7361

**Published:** 2020-03-22

**Authors:** Sofanit A Dessie, Deena Dahshan, Davinder Singh, Varun Dobariya, Sheena Pramod

**Affiliations:** 1 Internal Medicine, Marshall University, Joan C. Edwards School of Medicine, Huntington, USA; 2 Internal Medicine, Marshall University, Joan C Edwards School of Medicine, Huntington, USA; 3 Nephrology, Marshall University, Joan C Edwards School of Medicine, Huntington, USA

**Keywords:** hepatic abscess, pyogenic liver abscess, methicillin-resistant staphylococcus aureus (mrsa)

## Abstract

A hepatic abscess is a rare disease, especially in developed countries, and usually results from microbial contamination of liver parenchyma via an arterial or portal system or from a direct spread by contiguity. Pyogenic liver abscesses (PLA) are polymicrobial with Staphylococcus aureus accounting for less than 10% of the cases and methicillin-resistant Staphylococcus aureus (MRSA) accounting for even fewer. Colonic and hepatobiliary pathologies are often associated with reported MRSA abscesses. We report a case of MRSA bacteremia and liver abscess in an immunocompetent patient with no significant risk factors. Our patient presented with fever and abdominal pain of four days' duration. Laboratory studies revealed neutrophilic leukocytosis, elevated creatinine, c-reactive protein, and transaminitis. Blood culture was positive for MRSA. Computed tomography (CT) of the abdomen showed multiple areas of hypodensities over the left hepatic lobe that placed malignancy and abscess into the main differentials. A liver biopsy was consistent with liver abscess. Drainage was performed after a month of treatment with intravenous (IV) daptomycin and microbial analysis of the abscess was negative. Our case signifies the association of liver abscess and MRSA bacteremia in a patient with no significant risk factors and highlights the importance of prompt antibiotic treatment as first-line therapy.

## Introduction

Liver abscesses can have bacterial, fungal, or amoebic organisms as an etiology. Incidentally, amoebic and fungal abscesses occur predominantly in developing countries, mainly in Southeast Asia and Africa. Pyogenic liver abscesses (PLA) account for about 80% of all liver abscesses in developed countries and tend to be polymicrobial [1]. The microbiologic organisms involved in PLA include Escherichia coli and Klebsiella accounting for the majority of the cases, Staphylococcus aureus accounting for less than 10% of the cases, and methicillin-resistant Staphylococcus aureus (MRSA) with the lowest reported cases [2-3]. Risk factors associated with PLA include infections involving the hepatobiliary tract, colon, skin, and soft tissues [4]. Inflammatory bowel disease has also been reported to have an association with PLA because of the translocation of bacteria due to frequent portal venous bacteremia as a result of colonic disruption [5-6]. The mainstay of treatment of PLA is systemic antibiotics and surgical drainage of the abscess. Herein, we report the case of a liver abscess in an immunocompetent adult with community-acquired MRSA bacteremia that responded appropriately to systemic antibiotics before drainage, despite the large size of the abscess.

## Case presentation

**Figure 1:**
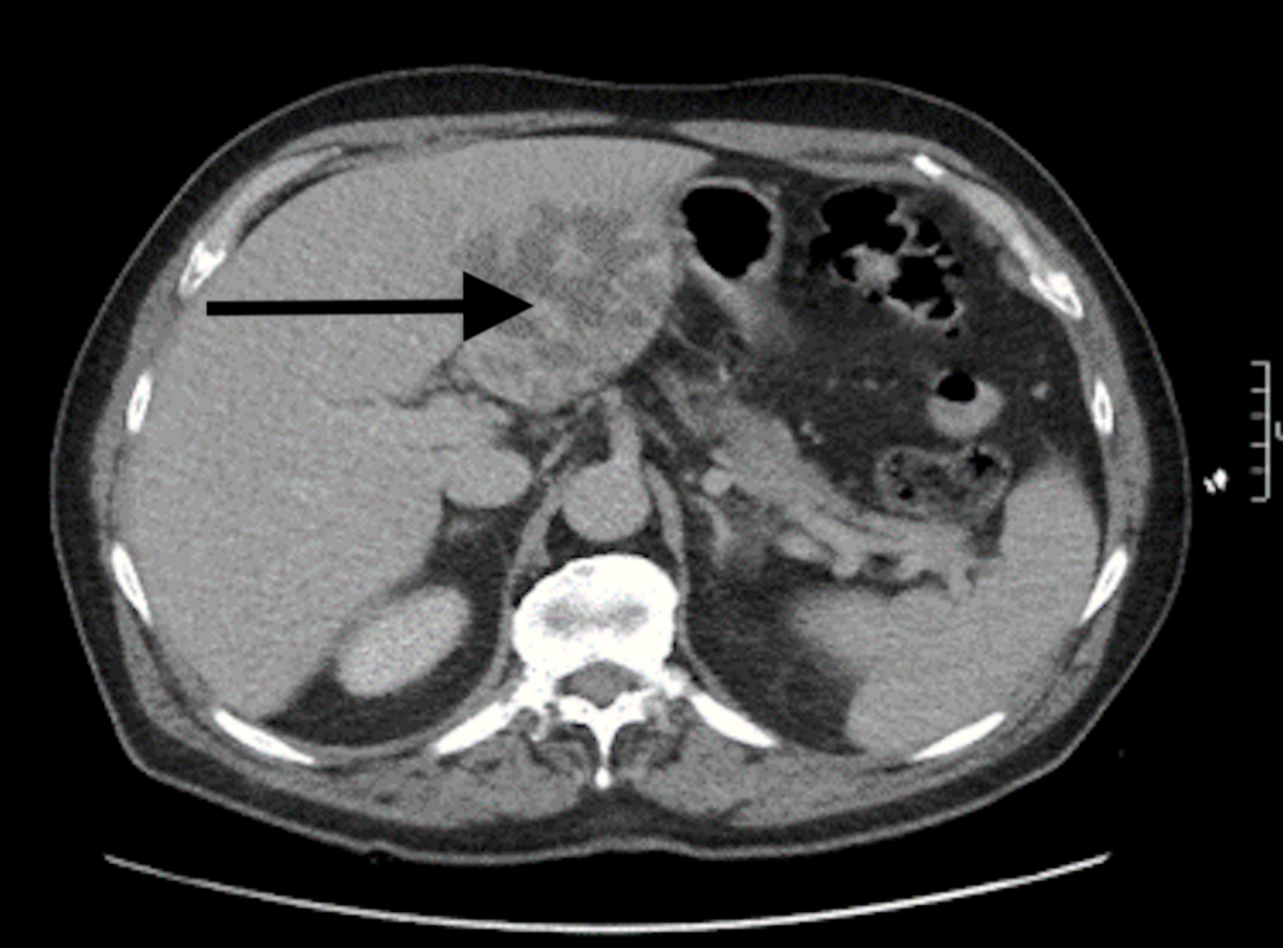
Axial computed tomography (CT) image of the abdomen showing a large, multi-loculated left hepatic abscess measuring 7.8 cm (as indicated by the arrow)

**Figure 2:**
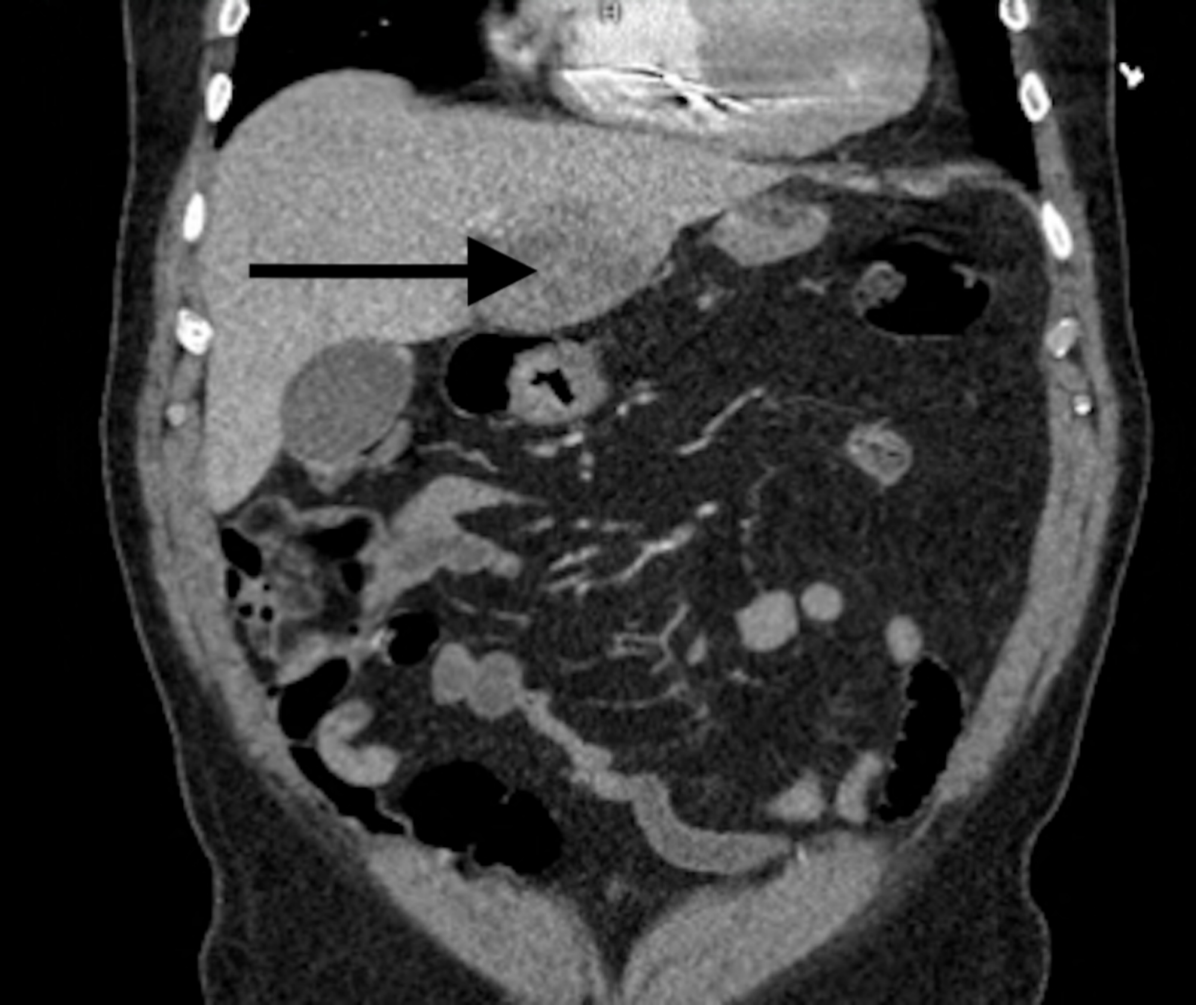
Coronal section of the abdomen showing the liver abscess on the left hepatic lobe as indicated by the arrow

A 73-year-old male with a past medical history significant for hyperlipidemia and atrial fibrillation with pacemaker implantation presented to the hospital with fever, abdominal pain, nausea, and decreased oral intake of four days' duration. He had no history of recent hospitalization, intravenous (IV) or subcutaneous drug use, or recent travel. The patient also had no surgical history other than the pacemaker implantation done three years before his presentation. Upon presentation, his vital signs were significant for a heart rate of 87 beats per minute, blood pressure of 81/42 mmHg (which responded to IV fluid administration), and a temperature of 39.2°C. Physical examination findings were unremarkable, except for dry buccal mucosa and mild generalized abdominal tenderness. Laboratory studies were significant for a white blood cell count of 16.7 x 109 /L (range: 4.5 to 11.0 x 109/L) with 84% neutrophils, creatinine of 1.8 mg/dL (range: 0.7 to 1.2 mg/dL), aspartate aminotransaminase of 103 U/L (range: 6 to 34 IU/L), alanine aminotransaminase of 81 U/L (range: 20 to 60 IU/L), total bilirubin of 1.3 mg/dL (range: 0.1 to 1.2 mg/dL), alkaline phosphatase of 79 U/L (range: 20 to 140 IU/L), C-reactive protein of 3.25 mg/L (range: below 3 mg/L), and erythrocyte sedimentation rate of 62 (range: 1 to 13 mm/hr). Urine drug screening was negative. MRSA was recovered from the blood. He was initially started on empiric vancomycin and piperacillin-tazobactam but the piperacillin-tazobactam was later discontinued after the final culture revealed MRSA bacteremia. Vancomycin was switched to daptomycin after three days due to worsening kidney function, and rifampin was added to protect the biofilm formation on the pacemaker. Transthoracic and transesophageal echocardiograms showed no evidence of infection around the pacemaker leads or vegetations. Computed tomography (CT) of the abdomen (Figures 1-2) revealed large complex masses with the left hepatic lobe measuring up to 7.8 cm and smaller hypodensities in the right hepatic lobes. A liver biopsy was performed, and the patient was discharged home to complete six weeks of IV antibiotics through a peripherally inserted central catheter. A complete resolution of his symptoms was reported on outpatient follow-ups. Repeat blood cultures after completion of antibiotics remained negative.

Liver biopsy pathology was consistent with hepatic abscess. Of note, the biopsy was done after two weeks of treatment with daptomycin. Due to the large size of the abscess, the patient underwent percutaneous abscess drainage four weeks after the initiation of antibiotics. Analysis from the hepatic abscess, including gram stain, routine bacterial, and amoebic serology, was negative. The source of his liver abscess was believed to be from MRSA bacteremia that was effectively treated with daptomycin before the abscess drainage which led to the negative bacterial culture of the liver abscess analysis.

## Discussion

Hepatic abscesses commonly occur following mucosal defects present on colonic lesions that allow a route for bacterial invasion into the portal system with subsequent hematogenous spread to the liver. Most pyogenic liver abscesses are polymicrobial, with enteric gram-negative bacilli and anaerobic species predominating. Staphylococcus aureus has been reported in less than 10% of liver abscess cases and the incidence of methicillin-resistant strain is even fewer.

Most of the case reports on MRSA liver abscesses are associated with underlying risk factors which include hepatobiliary and colonic pathologies, liver trauma, frequent hospitalization, and surgical procedures (Table 1) [7-11]. Upon literary search, we were able to find one case with a similar presentation as our patient that was reported in India [3]. There have been other fewer reported incidences of community-acquired MRSA liver abscesses in the literature, as outlined in Table 2 and were associated with potential predisposing factors, including immunocompromised state from chronic disease, skin infection, and incarceration [12-14]. In our patient, these risk factors were not present, and his liver abscess was considered to likely be community-acquired. The mortality rate from PLA can reach up to 15% as reported by Kuo et al. in 2013 in a series of 431 patients [15]. 

**Table 1:** Literature Review of Non-community-acquired MRSA Liver Abscesses

Author	Age	Gender	Risk associated or significant comorbidity	Country	Year published
Sloss et al. [7]	30	Male	Mechanical injury to the liver	Croatia	1995
Carilli et al. [8]	73	Male	frequent hospitalization	Turkey	1999
Shen et al. [4]	53	Female	Infected ventriculoperitoneal shunt	Taiwan	2003
van Vugt et al. [9]	18	Female	Navel piercing	Dutch	2005
Mancao et. al [10]	16	Female	Sickle cell disease	United States of America (USA)	2006
Albuquerque et. al. [6]	67	Male	Ulcerative colitis	Portugal	2011
Togashi et al. [5]	31	Male	Crohn’s disease	Japan	2013
Lezcano-Gort et al. [11]	76	Male	Colorectal cancer	Spain	2013

**Table 2:** Literature Review on Community-acquired MRSA Liver Abscesses USA: United States of America

Author	Age	Gender	Risk associated or significant comorbidity	Country	Year published
Chi et al. [12]	34	Male	End-stage renal disease, on dialysis	Taiwan	2004
Smith et al. [13]	24	Male	Skin infection prior to hepatic abscess	USA	2007
Wong V et al. [2]	25	Male	Prolonged antibiotic use for skin infection	China	2010
Cherian et al. [3]	81	Male	None - Community-acquired	India	2016
Igbinedion et al. [14]	21	Male	Prisoner	USA	2018
Our case	73	Male	None	USA	2020

With clinical signs of PLA and most laboratory investigations, including liver function tests, being non-specific, diagnosis essentially relies upon imaging. In our patient, a CT scan was performed which revealed large complex masses with the left hepatic lobe measuring up to 7.8 cm and smaller hypodensities in the right hepatic lobes. Treatment of PLAs has evolved over the last decade, such that percutaneous drainage is now the most common approach, with the potential to enhance systemic antibiotic therapy by providing bacterial speciation and antimicrobial sensitivities. Systemic antibiotic therapy alone has also been shown to be effective as the primary treatment of PLA in patients with small abscesses measuring less than 3 cm. A case study by Hope et al. reported a 100% success rate in a series of 107 patients treated with antibiotic therapy alone for hepatic abscesses measuring less than 3 cm [16]. However, CT-guided needle puncture with drainage is considered the first-line treatment of PLA [17]. 

In our case, the patient had clinical improvement with early antibiotic treatment before the CT-guided abscess drainage. This warrants further investigation as to the need for drainage in asymptomatic large-sized bacterial liver abscesses post-antibiotic treatment.

## Conclusions

This case illustrated an unusual etiology of MRSA-related, large size liver abscess in an otherwise immunocompetent host who underwent successful treatment with antibiotics prior to drainage. This raises an important question as to when drainage is absolutely indicated when patients are successfully treated with an antibiotic despite the large size of a liver abscess. 
